# IGFBP2 Is a Potential Master Regulator Driving the Dysregulated Gene Network Responsible for Short Survival in Glioblastoma Multiforme

**DOI:** 10.3389/fgene.2021.670240

**Published:** 2021-06-15

**Authors:** Manasa Kalya, Alexander Kel, Darius Wlochowitz, Edgar Wingender, Tim Beißbarth

**Affiliations:** ^1^Department of Medical Bioinformatics, University Medical Center Göttingen, Göttingen, Germany; ^2^geneXplain GmbH, Wolfenbüttel, Germany; ^3^Institute of Chemical Biology and Fundamental Medicine SB RAS, Novosibirsk, Russia

**Keywords:** glioblastoma, master regulators, upstream analysis, IGFBP2, FRA-1, FOSL1, short term survivors, transcription factors

## Abstract

Only 2% of glioblastoma multiforme (GBM) patients respond to standard therapy and survive beyond 36 months (long-term survivors, LTS), while the majority survive less than 12 months (short-term survivors, STS). To understand the mechanism leading to poor survival, we analyzed publicly available datasets of 113 STS and 58 LTS. This analysis revealed 198 differentially expressed genes (DEGs) that characterize aggressive tumor growth and may be responsible for the poor prognosis. These genes belong largely to the Gene Ontology (GO) categories “epithelial-to-mesenchymal transition” and “response to hypoxia.” In this article, we applied an upstream analysis approach that involves state-of-the-art promoter analysis and network analysis of the dysregulated genes potentially responsible for short survival in GBM. Binding sites for transcription factors (TFs) associated with GBM pathology like NANOG, NF-κB, REST, FRA-1, PPARG, and seven others were found enriched in the promoters of the dysregulated genes. We reconstructed the gene regulatory network with several positive feedback loops controlled by five master regulators [insulin-like growth factor binding protein 2 (IGFBP2), vascular endothelial growth factor A (VEGFA), VEGF165, platelet-derived growth factor A (PDGFA), adipocyte enhancer-binding protein (AEBP1), and oncostatin M (OSMR)], which can be proposed as biomarkers and as therapeutic targets for enhancing GBM prognosis. A critical analysis of this gene regulatory network gives insights into the mechanism of gene regulation by IGFBP2 *via* several TFs including the key molecule of GBM tumor invasiveness and progression, FRA-1. All the observations were validated in independent cohorts, and their impact on overall survival has been investigated.

## Introduction

Glioblastoma multiforme (GBM) is the most common, highly malignant primary brain tumor (Wen and Kesari, 2008). Despite huge developments in treatment strategies, GBM poses unique treatment challenges due to tumor recurrence (34%) and drug resistance leading to poor survival rates of less than 15 months even after advanced chemoradiotherapy (Krex et al., 2007). As few as 2% of patients respond to standard therapy and survive beyond 36 months (Krex et al., 2007; Das et al., 2011), clinically called long-term survivors (LTS). Another group termed short-term survivors (STS) are those who survive less than 12 months (Shinawi et al., 2013). The factors that determine the long survival are not well understood.

Though several factors like age, gender, Karnofsky Performance Score, the extent of tumor resection, radiotherapy, and chemotherapy are associated with survival and treatment response (Scott et al., 1999; Lee et al., 2008; Sonoda et al., 2009; Zhang et al., 2012), it is evident from recent research that certain molecular signatures can be connected with treatment response and thereby survival. Promoter methylation of the gene MGMT, mutations in the genes IDH1/2, and loss of heterozygosity in chromosome 1p/19q have been confirmed to be highly informative (Krex et al., 2007; Das et al., 2011; Zhang et al., 2012; Han et al., 2014; Reifenberger et al., 2014; Franceschi et al., 2015; Chen et al., 2016). Furthermore, CHI3L1, FBLN4, EMP3, IGFBP2, IGFBP3, LGALS3, MAOB, PDPN, SERPING1, and TIMP1 gene expression has repeatedly been reported to be decreased in LTS patients (De Vega et al., 2009; Bi and Beroukhim, 2014; Han et al., 2014; Franceschi et al., 2015). A better characterization of these extreme survival groups at the molecular level will likely shed important light on the biological aspects that drive their malignancy and survival.

With the advent of gene expression profiling and remarkable developments in high-throughput technologies, it is possible to gain deeper molecular insights into disease biology. Databases like Gene Expression Omnibus—GEO (Barrett et al., 2013), Array Express (Athar et al., 2019), and The Cancer Genome Atlas—TCGA (Grossman et al., 2016) serve as open platforms for retrieval of high-quality multi-omics data to search for new markers in cancer research. The analysis of differentially expressed genes (DEGs) is already an important and established *in silico* strategy to identify potential drivers of cellular state transitions. For a more refined analysis, annotation of DEGs, using *a priori* known biological categories from the Gene Ontology (GO; Ashburner et al., 2000) and pathway databases, e.g., TRANSPATH^®^ (Krull et al., 2003), KEGG (Kanehisa et al., 2020), PANTHER (Thomas et al., 2003), and Reactome (Jassal et al., 2020), has proven to be an effective hypothesis-driven approach in cancer research. Moreover, with the advent of state-of-the-art promoter analysis, it is now possible to establish gene regulatory networks computationally that can be used to understand the causes of gene dysregulation and for identification of causal master regulators driving them. In this regard, we applied the Genome Enhancer^1^, a multi-omics analysis tool that makes use of the open-source programming environment BioUML (Kolpakov et al., 2019) and incorporates an automated pipeline for the previously published “upstream analysis” (Koschmann et al., 2015; Boyarskikh et al., 2018) and the “walking pathways” (Kel et al., 2019) approach. There are two major steps that constitute this strategy: (1) analysis of the promoters of DEGs to identify relevant transcription factors (TFs): this is done with the help of the TRANSFAC^®^, database (Matys et al., 2006) and the binding site identification algorithms, MATCH^TM^ (Kel et al., 2003, 2006) and CMA (Waleev et al., 2006); (2) reconstruction of signaling pathways that activate these TFs and identification of master regulators on the top of such pathways: for this, the signaling pathway database TRANSPATH^®^, (Krull et al., 2003) has been employed in conjunction with special graph search algorithms that identify positive feedback loops (Kel et al., 2019).

In this study, we applied the upstream analysis to publicly available datasets of GBM from the GEO database to understand the gene-regulatory networks contributing to short survival in GBM. This regulatory network revealed a set of 12 TFs binding to the regulatory regions of the genes of interest and five master regulators regulating them, namely, (a) vascular endothelial growth factor A (VEGFA), a mediator of angiogenesis (Xu et al., 2013) and a promoter of stem-like cells in GBM; (b) PDGF, a highly amplified gene and key player of tumorigenesis (Martinho and Reis, 2011); (c) oncostatin M (OSMR), which orchestrates feed-forward signaling with EGFR and STAT3 to regulate tumor growth (Jahani-As et al., 2016); (d) adipocyte enhancer-binding protein (AEBP1), which plays a key role in pathogenesis through NF-κB activation (Majdalawieh et al., 2020); and (e) IGFBP2.

Insulin-like growth factor binding protein 2, a well-established molecule of interest in GBM (Yao et al., 2016), was found to be more highly expressed in STS and to have an impact on overall survival. IGFBP2 expression is said to be higher in all four (classical, mesenchymal, proneural, and neural) GBM subtypes (Lindström, 2019). It also drives gene programs for immunosuppression in the mesenchymal subtype and is suggested as an immunotherapeutic target (Liu et al., 2019). In non-mesenchymal subtypes (classical, proneural, and neural), it modulates cell proliferation (Phillips et al., 2016; Cai et al., 2018). It has also been found to be a marker of tumor aggressiveness and a prognostic marker for survival (Lindström, 2019). However, the molecular mechanism by which IGFBP2 affects disease progression and patient prognosis is not fully understood.

This work focuses on understanding gene regulatory networks that drive short survival in GBM and their master regulators, which we suggest as biomarkers and therapeutic targets. Later, we critically discuss the role of IGFBP2 in the gene regulatory network.

## Results

### Identification of Differentially Expressed Genes

Identifying DEGs gives us insight into the biological semantics of a cellular state and helps to identify promising biomarkers of various disease states. The differential gene expression analysis between STS and LTS groups of GBM, from the batch-corrected GSE dataset, was performed using linear models for microarray data (LIMMA) (Ritchie et al., 2015) with FDR cutoff of 5%. The analysis revealed 957 genes that are significantly differentially expressed (DEGs) (adjusted *p*-value < 0.05). Furthermore, the analysis revealed 115 significantly upregulated (log2FC > 0.5) and 83 significantly downregulated [log2FC < (−0.5)] genes. The top five upregulated and downregulated genes and their corresponding log2FC are shown in Table 1 and the full list is given in Supplementary Table 1-A.

**TABLE 1:** The list of the top five significantly upregulated and downregulated genes in STS identified in the GSE dataset.

Gene symbol	Description	Log2FC	*p*-Value	Adjusted *p*-value
**Upregulated genes**				
CHI3L1	Chitinase-3-like 1	1.371	9.73E−05	0.013
PDPN	Podoplanin	1.241	7.88E−07	0.002
MEOX2	Mesenchymal homeobox 2	1.159	6.45E−04	0.028
IGFBP2	Insulin-like growth factor binding protein 2	1.149	4.87E−05	0.010
COL6A2	Collagen type VI alpha 2 chain	1.0479	5.79E−05	0.011
**Downregulated genes**				
KLRC2	Killer cell lectin-like receptor C2	−1.2187	3.63E−04	0.022
KLRC1	Killer cell lectin-like receptor C1	−1.2187	3.63E−04	0.022
FUT9	Fucosyltransferase 9	−1.0709	1.15E−04	0.014
DPP10	Dipeptidyl peptidase-like 10	−1.02781	2.97E−05	0.008
GABRB3	Gamma-aminobutyric acid type A receptor subunit beta3	−0.96352	6.73E−05	0.011

### Functional Annotation of Differentially Expressed Genes

**FIGURE 1:**
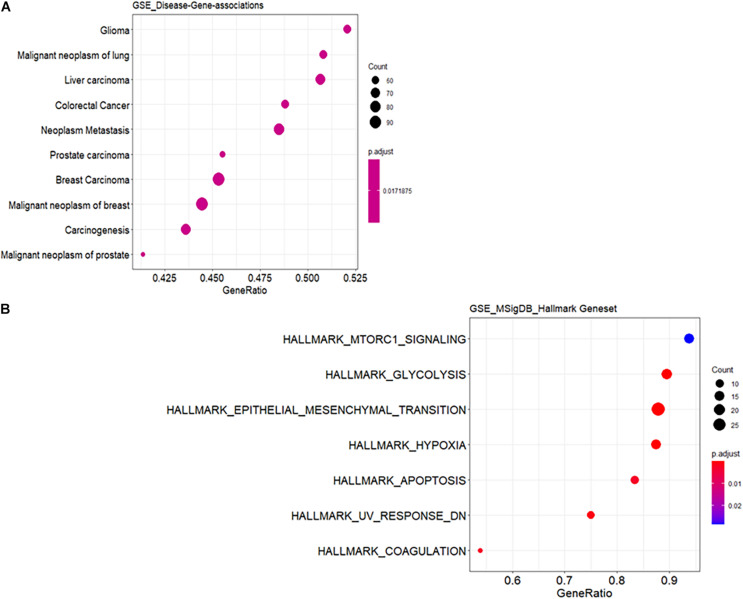
Functional enrichment analysis of differentially expressed genes (DEGs). **(A)** Enrichment for known disease gene networks in different diseases. *Y*-axis represents enriched ontology categories and *X*-axis represents gene ratio. Gene ratio is count/set size. The “count” is the number of genes that belong to a given gene set, while “set size” is the total number of genes in the gene set. *Y*-axis is sorted based on leading edge. Leading edge is a subset of genes that contributes most to the enrichment score. The dots are sized based on gene ratio and are colored according to their adjusted *p-*value. **(B)** Enrichment for hallmark gene sets in the molecular signature database similar to **(A)**.

Functional annotation was performed to investigate the biological roles of these DEGs. As shown in Supplementary Figure 1A, the top GO biological processes are extracellular structure and matrix organization with 30 DEG hits. Supplementary Figure 1B shows the results for GO cellular component enrichment, which revealed dysregulation of genes that encode proteins for the extracellular matrix and synaptic membranes. The important enriched molecular function GO terms are channel activity and transmembrane transporter activity (Supplementary Figure 1C). The disruption in extracellular matrix organization is one of the important signatures in glioblastoma treatment response dealing with invasiveness and malignancy (De Vega et al., 2009). Deeper biological insights are required in this aspect. It is interesting to see enrichment of genes known to be involved in glioma (Figure 1A). Gene signature enrichment based on hallmark gene sets of MSigDB clearly signifies the enrichment of epithelial-to-mesenchymal transition depicted in Figure 1B. The process of epithelial-to-mesenchymal transition plays a very important role in GBM survival by driving tumor invasiveness and drug resistance (Iwadate, 2016). Important pathways like Aurora signaling, G2/M phase transition, and TGF-β pathway are found to be enriched according to TRANSPATH^®^, (Table 2). The full list of enrichment results can be found in Supplementary Table 1-B.

**TABLE 2:** Pathway enrichment using the TRANSPATH^®^, pathway (2019.3) for differentially expressed genes.

ID (TRANSPATH)	Title	Group size	Expected hits	Nominal *p-*value	ES	Rank at max	NES	FDR	Number of hits
CH000001004	Aurora-A cell cycle regulation	68	67.262	0	0.422	8,347	4.138	0	68
CH000000919	Cyclosome regulatory network	77	76.164	0	0.349	7,336	3.728	0	77
CH000000694	G2/M phase (cyclin B: Cdk1)	66	65.284	0	0.375	6,641	3.587	0	66
CH000000879	Caspase network	83	82.099	0	0.333	8,414	3.523	0	83
CH000000711	TGFbeta pathway	153	151.340	0	0.232	8,431	3.346	0	151

### Identifying the Master Regulators of Dysregulated Gene Networks

Reconstruction of the disease-specific regulatory networks can help to identify potential master regulators that may serve as mechanism-based biomarkers or as therapeutic targets to block a specific pathological regulatory cascade. Using the promoter analysis as a first step, we analyzed enrichment of TF binding sites in promoters of upregulated genes of STS using DNA-binding motifs from the TRANSFAC^®^, library. Two hundred seventy-four TFs (Supplementary Table 1-C) enriched for CCKR signaling, interleukin signaling, PDGF signaling, and WNT signaling were found to have their binding sites enriched; full enrichment results can be found in Supplementary Table 1-D.

Next, we applied the Composite Module Analyst (CMA) and identified two modules involving 12 TF binding site combinations that regulate the expression of the genes of interest. CMA revealed the following modules comprising clustering binding sites for the following TFs: module 1: HNF3B, NANOG, NFKAPPAB, TAF1, TCF4, and FRA-1; module 2: PPARG, TAL1, REST, POU6F1, FOSJUN, and PBX. The modules and their significance are depicted in Supplementary Figure 2. Differential expression statistics for the 12 TFs are given in Supplementary Table 2. Among them, FRA-1 TF (also known as FOSL1) was found to be *p*-value significant and upregulated in STS of GBM (log2FC = 0.023, *p-*value = 0.008, adjusted *p-*value (0.093) (Supplementary Table 2).

**FIGURE 2:**
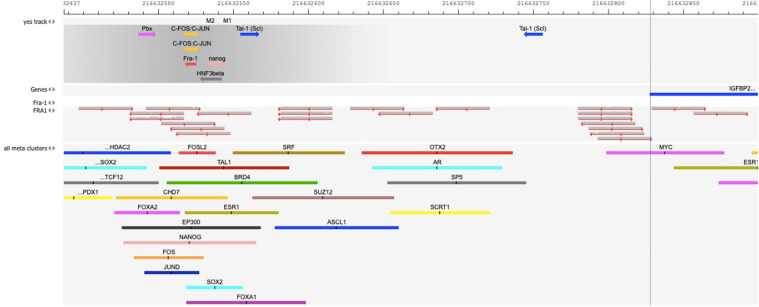
Map of the cluster of transcription factor (TF) binding sites of the composite model identified within the promoter of IGFBP2 gene [–1000 to + 100 bp relative to TSS]. The position of the TSS (the beginning of the first exon on IGFBP2 gene) is shown by the vertical dotted line. “Yes track” represents the cluster of identified TF binding sites of the composite model within the promoter. The direction of the arrows gives the orientation of the PWMs. The names of TFs binding to these sites are shown above the arrows. The track “FRA1” represents the mapped reads of the FRA1 (also called FOSL1) ChIP-seq data of GEO, GSM803382. The reads were mapped on the hg38 human genome using Subread aligner (Liao et al., 2013) with default parameters. The track “all meta clusters” shows all known meta-clusters in this region from the GTRD database that represent the overlapping fragments of peaks for one particular TF from several ChIP-seq experiments. The name of TF is shown above each meta-cluster. Several predicted TF binding sites in the composite model are confirmed in independent ChIP-seq experiments: several overlapping reads of FRA1 ChIP-seq data in the “FRA1” track and FOSL2 meta-cluster in the GTRD confirm the predicted site for Fra-1; FOS and JUN meta-clusters in the GTRD confirm the predicted c-Fos/c-Jun binding sites; NANOG meta-cluster confirms the predicted Nanog binding site; TAL1 meta-cluster confirms the predicted Tal-1 binding site; FOXA2 and FOXA1 meta-clusters of the GTRD confirm the HNF3beta binding site.

Figure 2 validates the predicted cluster of TF binding sites from the composite modules identified in the promoter of IGFBP2 gene. We can see that binding sites for the TFs – c-Fos/c-Jun, Nanog, Tal-1, and HNF3/FoxA1 in this cluster can be confirmed by publicly available ChIP-seq data of the GTRD database (Kolmykov et al., 2021). In addition, binding site of FRA-1 can be confirmed by a cluster of mapped reads of independent publicly available ChIP-seq data (FRA1 track in Figure 2) (full map is shown in the Supplementary Figure 4).

**FIGURE 3:**
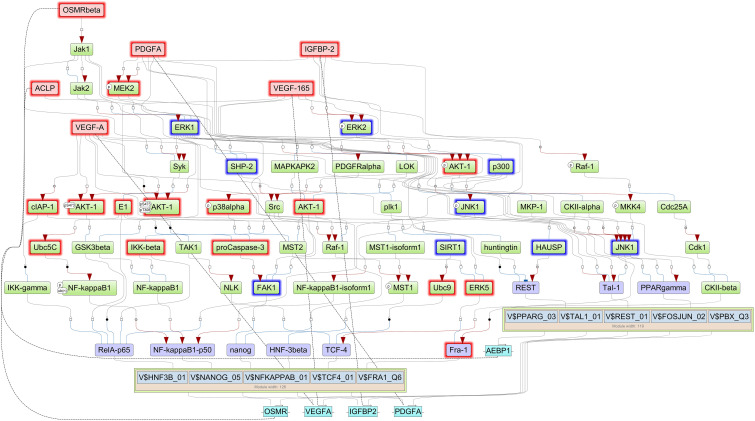
Signal transduction and gene regulatory network of six master regulators (red nodes) regulating two transcription factor modules (purple nodes) enriched in promoters of highly upregulated genes of short-term survivors (STS). The dotted lines from genes to such signaling proteins represent the transcription and translation processes (positive feedback loops). The outside box filling is based on log2FC < (−0.2) and is filled red when upregulated (log2FC > 0.2 and *p*-value < 0.05) and filled blue when downregulated (log2FC < 0.2 and *p*-value < 0.05) in the current study.

Finally, we reconstructed signaling network that activates the TFs revealed by CMA analysis and thereby identifying the top regulators in these networks using the TRANSPATH^®^, database. With this approach, we identified five important master regulators that are plausible drivers of short survival in GBM: IGFBP2, VEGFA/VEGF165, platelet-derived growth factor A (PDGFA), AEBP1, and OSMR. All the master regulators were found to be significantly upregulated in STS. The genes that encode the master regulator proteins are controlled by the TFs revealed by CMA in their promoters, which maintains the multiple positive feedback loops in the system. It should be underlined here that, in such networks with positive feedback loops, the identified key TFs, such as FRA-1, are both upstream of their target genes, among them the IGFBP2, as well as downstream from the master regulator proteins, one of them the IGFBP2 protein. The regulatory network reconstructed with six master regulators is shown in Figure 3, and the master regulators and their log2FC in STS are listed in Table 3. Since VEGF165 is a splice variant of VEGFA, only the latter will be considered further on.

**TABLE 3:** Table of the master regulators identified, their description, log2FC in STS, and number of transcription factors regulated.

Molecule name	Gene description	HGNC gene symbol	Log2FC in STS	Number of TFs regulated
IGFBP2	Insulin-like growth factor binding protein 2	IGFBP2	1.149	9
ACLP	AE-binding protein 1	AEBP1	0.782	9
VEGFA	Vascular endothelial growth factor A	VEGFA	0.778	9
VEGF165	Vascular endothelial growth factor A	VEGFA	0.778	9
OSMRbeta	Oncostatin M receptor	OSMR	0.634	8
PDGFA	Platelet-derived growth factor subunit A	PDGFA	0.529	9

### Validating the Expression of Master Regulators in Other Cohorts

The expression patterns of the master regulators identified above have been validated in two different cohorts: (A) TCGA-GBM microarray data (Grossman et al., 2016) and (B) GSE16011 (Gravendeel et al., 2009). The expression patterns were similar, and there is a significant upregulation of all master regulators except for VEGFA (GSE16011: adjusted *p-*value = 0.069 and TCGA-GBM: adjusted *p-*value = 0.075) (Supplementary Tables 1-E, 1-F). The differential expression values are given in Table 4.

**TABLE 4:** Expression of the master regulators identified across survival groups (STS and LTS, respectively) and across three datasets (GSE,GSE16011 and TCGA-GBM microarray).

Master regulator	GSE		GSE16011		TCGA	
	Log2FC (STS vs LTS)	Adjusted *p-*value	Log2FC (STS vs LTS)	Adjusted *p-*value	Log2FC (STS vs LTS)	Adjusted *p-*value
IGFBP2	1.149	4.87E−05	2.030	4.598E−04	1.098	5.00E−06
AEBP1	0.782	7.75E−05	1.723	0.001	0.971	3.96E−06
PDGFA	0.529	4.55E−04	1.680	4.709E−09	0.825	2.07E−05
VEGFA	0.778	5.20E−04	0.884	0.069	0.500	0.0752
OSMR	0.634	8.65E−04	1.957	4.24E−05	0.486	0.0318

### Validating the Master Regulators in the TCGA-GBM Cohort

The TCGA-GBM microarray data containing 271 STS and 49 LTS is used to validate the above-identified drivers of short survival. The data is preprocessed and adjusted for batch effects (Supplementary Figure 3), and a differential gene expression analysis is performed. Same cutoffs for log2FC and adjusted *p-*value are used. We identified 171 genes upregulated in STS of GBM (log2FC > 0.5 and adjusted *p-*value < 0.05) (full list in Supplementary Table 1-E). Forty-nine of them were in common between the GSE dataset and TCGA-GBM; the full differential gene expression analysis results are given in Supplementary Table 1-G. Composite models selected by the CMA algorithm across the two datasets were expected to vary. We identified a model that includes a set of 16 TFs (Supplementary Table 3) and 12 master regulators upstream of them (Supplementary Table 4) regulating the signal transduction and gene regulatory network in STS.

As a result, the TCGA-GBM dataset validates IGFBP2, AEBP1 (ACLP), and PDGFA as master regulators driving the dysregulated gene network in STS. We also found that binding sites for FRA-1 TF are statistically significantly enriched at the regulatory regions of the dysregulated genes including IGFBP2 in the TCGA-GBM cohort (Supplementary Table 5).

### Impact of Master Regulators on Survival in GBM

**FIGURE 4:**
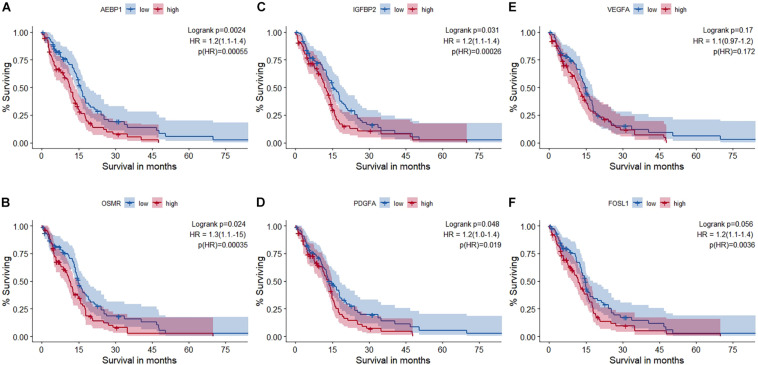
Survival analysis using RNA-seq data of The Cancer Genome Atlas glioblastoma (TCGA-GBM) cohort. **(A)** AEBP1, **(B)** OSMR, **(C)** IGFBP2, **(D)** PDGFA, **(E)** VEGFA, and **(F)** FRA-1 (FOSL1). Out of the five master regulators, all but VEGFA (AEBP1, OSMR, PDGFA, and IGFBP2) had a statistically significant impact on survival. Hazard ratio (HR) and statistical significance [*p* (HR)] according to Cox survival estimates are mentioned.

Univariate survival analysis was used to study the impact of these master regulators and the TFs they regulate on the overall survival in GBM based on TCGA-RNA-seq data. Patients are split into non-overlapping 50% upper and lower quantiles. Additionally, cox regression for the univariate survival analysis is performed, and hazard ratio (HR) and corresponding *p-*value*s* are shown in Figure 4. Univariate survival Cox regression analysis on other microarray datasets is given in Supplementary Table 1-I. All master regulators were found to have a significant impact upon survival except VEGFA. FRA-1 (FOSL1) was found to have a significant HR.

### Master Regulator Expression Patterns Across GBM Subtypes

Based on the regulatory landscape of GBM, there are four subtypes—classical, mesenchymal, proneural, and neural (Verhaak et al., 2010). There is a significant level of intertumoral as well as intra-tumoral heterogeneity within each of them (Verhaak et al., 2010; Bradshaw et al., 2016). Molecular subtypes of GBM in the GSE dataset is given in Supplementary Table 6. DEGs between STS and LTS within each subtype are given in Supplementary Table 7. The expression patterns of master regulators across subtypes and across survival groups are depicted as boxplot in Supplementary Figure 5. None of the master regulators were found to be significantly differentially expressed between survivor groups in any subtypes.

## Discussion

Gene regulatory networks represent the causal regulatory relationships between TFs and their gene targets, which enables us to discover dysregulated genes in certain biological states (Marbach et al., 2012). Comparative studies of STS and LTS of GBM showed that gene expression programs executed across survival groups vary significantly. In the light of these findings, we sought to apply an upstream analysis approach to gain an insight about gene regulatory networks driving the short survival.

In the promoter analysis, we identified a set of 12 TFs in composite clusters that are enriched in the promoter regions of dysregulated genes in STS (upregulated in STS). For several of these TFs, a connection to GBM has previously been established. The TFs NANOG and REST are critical for self-renewal and maintenance of oncogenic signatures in glioblastoma stem-like cells (Kamal et al., 2012; Bradshaw et al., 2016); PPARG has emerged as a promising therapeutic target as its agonists increased median survival in GBM patients (Ellis and Kurian, 2014); NF-κB is implicated in several processes like invasion, epithelial–mesenchymal transition (Yamini, 2018), resistance to radiotherapy (Avci et al., 2020), and maintenance of cancer stem-like cells (da Hora et al., 2019); and FRA-1/FOSL1 has been reported to be important in maintenance/progression of malignant glioma (Debinski and Gibo, 2005). FRA-1 along with JUN-B modulates a malignant feature of GBM by regulating the expression of the metalloproteinases like MMP-2 and MMP-9 (Kesari and Bota, 2011). Among these 12 TFs, we found that FRA-1 has a significant impact upon survival and has a higher expression in STS. Debinski and Gibo (2005) hypothesized that any AP1-stimulating signals like epidermal growth factor (EGF), leukemia inhibitory factor, OSMR, or FGF-2 can positively regulate FRA-1. VEGF-D is regulated by FRA-1 (supporting the feedback loop found in our work) and is a known prognostic factor in other aggressive cancers (Debinski et al., 2001; Azar et al., 2014).

A graph analysis of the signal transduction network upstream of these TFs identified five potential master regulators that might explain gene dysregulation in STS, namely, insulin-like growth factor binding protein 2 (IGFBP2), VEGFA, its isoform VEGF165, PDGFA, OSMR, and AEBP1. All the identified master regulators were upregulated in STS, and their expression patterns were validated computationally in two other independent cohorts. We found that the expression of all master regulators, with the exception of VEGFA, was correlated with overall survival in the GBM patients. IGFBP2, AEBP1, and PDGFA master regulators driving short survival were validated as master regulators of short survival in the TCGA-GBM microarray cohort. Out of them, IGFBP2 had higher expression in STS. The IGFBP2 is said to be one of most potential glioma oncogenes and functions as a hub of oncogenic signaling pathways by regulating pro-tumorigenic signals of tumor initiation and progression. Earlier studies have suggested IGFBP2 to drive EMT and as a potential therapeutic target in mesenchymal GBM (Yamini, 2018; Liu et al., 2019). It is established that exogenous IGFBP2 promotes proliferation, invasion, and chemoresistance to temozolomide in glioma cells *via* integrin β1 by promoting ERK phosphorylation and nuclear translocation (Schütt et al., 2004; Yau et al., 2015). IGFBP2 is considered as one of the strongest biomarkers of aggressive behavior in GBM (Holmes, 2012; Phillips et al., 2016) and also a prognostic marker for survival (McDonald et al., 2007; Phillips et al., 2016).

**FIGURE 5:**
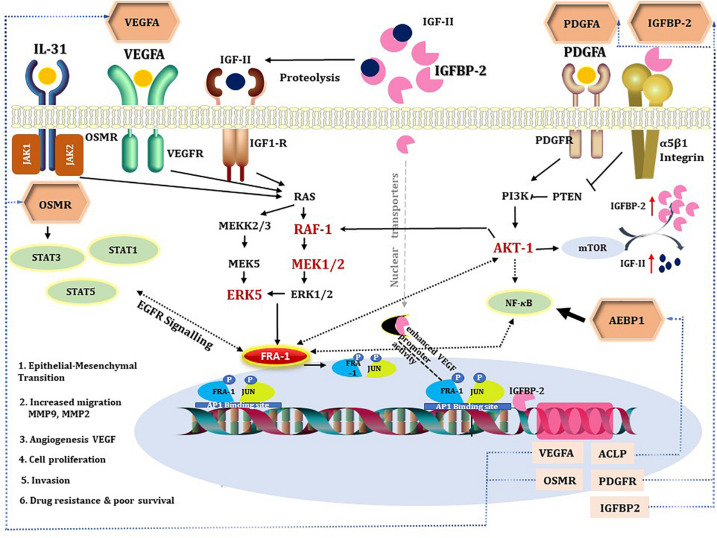
A diagram combining prior knowledge about the role of IGFBP2 in GBM and the gene regulatory network developed in the study. The hexagons are master regulators identified in our analysis. All the intermediates of the gene regulatory network are colored red if upregulated in STS and black if not present in the network. Dotted black line indicates the knowledge is through literature and continuous black line if known through gene regulatory network. Blue dotted lines represent gene regulatory connections between master regulators and their corresponding genes transcribed by target transcription factors. VEGFA, PDGFA, IGFs, and IL-31 activate RAF/MEK/ERK signaling, which mediate cell survival through PI3K-AKT pathway (Yao et al., 2016; Simpson et al., 2017). MEK2/RAF1/ERK5 and AKT-1 are found to be upregulated in STS, suggestive of activated ERK signaling, which can contribute to drug resistance (Abrams et al., 2010; Salaroglio et al., 2019). IGFBP2 activates IGFR either by increasing bioavailability of IGFs or by direct interaction with its functional domain. Integrin acts as receptor for IGFBP2 extracellular signals (Schütt et al., 2004; Yau et al., 2015) and modulates NF-κB signaling. IGFBP2 by nuclear translocation (Azar et al., 2011) is involved in transcriptional regulation of the VEGF gene and modulates angiogenesis (Azar et al., 2011). STAT3 and NF-κB are said to be the two major downstream transcription factors of IGFBP2 that direct tumorigenic intracellular signaling (Phillips et al., 2016) *via* EGFR signaling. Oncostatin M, a receptor for cytokine IL31, is a regulator of EGFR signaling (Jahani-As et al., 2016). FRA-1 is required for AKT activation in cancers to promote AKT-dependent cell growth (Zhang et al., 2016). NF-κB can regulate AP1 (FOS and JUN) thereby VEGF expression in pancreatic tumor cell lines (Fujioka et al., 2004). All the five master regulators have binding sites for FRA-1. In the figure, we depicted the possible positive feedback loop between FRA-1 and the master regulators to orchestrate a complex tumorigenic program of invasiveness, migration, drug resistance, and angiogenesis.

Here, we propose that IGFBP2 can be a potential regulator of FRA-1 TF. IGFBP2-induced RAF/MAPK signaling can activate FRA-1 (Figure 3). It has been shown earlier that IGFBP2 and FRA-1 regulate transcription of VEGF (Debinski et al., 2001; Azar et al., 2011, 2014), which is the second most dysregulated master regulator in our network. Enhanced ERK signaling, triggered by these master regulators, may lead to mitogen-induced FRA-1 transcription (Adiseshaiah et al., 2005) as well as its protection from proteasomal degradation (Vial and Marshall, 2003). The gene regulatory network deduced here suggests that FRA-1 mediates a positive feedback loop where it activates transcription of master regulator genes in cooperation with other TFs, which in turn cause an increase in FRA-1 activity. Promoters of the genes of all five master regulators reported in the study contain potential binding sites for FRA-1. Experimental evidences that IGFBP2 can drive GBM invasion by enhancing MMP2 expression (Wang etal., 2003) support our computational prediction of IGFBP2 as a therapeutic target. Hence, the gene regulatory networks proposed by our computational analysis suggest a novel molecular mechanism associated with GBM survival in which FRA-1 acts as a transcription regulator of IGFBP2. The study of Kesari and Bota (2011) confirmed our hypothesis that IGFBP2 can enhance GBM invasion *via* TF AP1 (FOS-JUN). Metalloproteinases like MMP-2/MMP-9 have been reported earlier to be regulated by FRA-1 in several cancers including GBM (Debinski and Gibo, 2005; Adiseshaiah et al., 2008; Kimura et al., 2011; Prywes and Henckels, 2013). Taking these findings together, our work proposes that the regulation of IGFBP2 gene expression *via* AP1 (FOS-JUN) can be an important mechanism of GBM invasion. An overview of the gene regulatory network developed in this work and supporting literature evidence is illustrated in Figure 5.

In summary, our work proposes a gene regulatory network associated with STS in GBM, which is regulated by five master regulators, namely, IGFBP2, VEGFA, PDGFA, OSMR, and AEBP1. Furthermore, these five master regulators may present biomarkers of GBM prognosis and/or as therapeutic targets for enhancing survival in GBM. This work also proposes a novel mechanism of gene dysregulation by IGFBP2 by modulating a key molecule of tumor invasiveness and progression—FRA-1 TF. All the genes encoding these five master regulators have binding sites for FRA-1 in their promoters. FRA-1 and the master regulators cooperate in a positive feedback loop to orchestrate a complex tumorigenic program leading to poor survival in GBM.

## Materials and Methods

### Data Collection

The genome-wide expression profiles based on Human Genome U133 plus 2.0 array and clinical information of patients with GBM were collected from the public repository of GEO database—GSE108474 (Gusev et al., 2018)^2^ and GSE53733 (Reifenberger et al., 2014)^3^. The two datasets were pooled together leading to 113 and 58 samples corresponding to STS (survival <12 months) and LTS (survival >36 months) with GBM, respectively (Table 5). Duplicates were not removed. Sample information and cleaned datasets are given in GitHub.

**TABLE 5:** Statistics of datasets under study.

	Platform	Short-term survivors	Long-term survivors
GSE53733 (Reifenberger et al., 2014)	HU133 plus 2.0 arrays	16	23
GSE108474 (Gusev et al., 2018)	HU133 plus 2.0 arrays	97	35

*The datasets with labels GSE’ were collected from the GEO database.*

### Affymetrix Microarray Data Pre-processing

The raw data files (.CEL format) for GSE108474 and GSE53733 were collected from the GEO database—from here on called as GSE dataset. RMA algorithm is used in R (affy package) for background correction, quality check, and normalization to obtain log2-transformed expression values (Gautier et al., 2004). Batch correction of the pooled expression data was performed using empirical Bayes framework (Leek et al., 2012). This batch-corrected file is used for further analysis. Multiple Affymetrix IDs were summarized to gene IDs by choosing the maximum out of the probe intensities of multiple probes belonging to a single gene. The final expression matrix comprised 21,526 probes and 171 samples.

### Differential Gene Expression Analysis

The LIMMA method was applied to identify DEGs (Ritchie et al., 2015). It is an efficient tool that is stable even for experiments with small samples. A differential gene expression analysis of 171 samples of the GSE dataset was performed with Benjamini–Hochberg adjusted *p*-value. Nine hundred fifty-seven genes were significantly (adjusted *p-*value < 0.05) differentially expressed (DEGs). One hundred fifteen of them were significantly upregulated (adjusted *p-*value < 0.05 and log2FC > 0.5) and 83 were significantly downregulated [adjusted *p-*value < 0.05 and log2FC < (−0.5)].

### Databases Used in the Study

Transcription factor binding sites in promoters and enhancers of DEGs were analyzed using known DNA-binding motifs described in the TRANSFAC^®^, library, release 2019.3 (geneXplain GmbH, Wolfenbüttel, Germany)^4^ (Wingender et al., 1996). The master regulator search uses the TRANSPATH^®^, database, release 2019.3 (geneXplain GmbH, Wolfenbüttel, Germany)^5^ (Krull et al., 2003). A comprehensive signal transduction network of human cells is built by the Genome Enhancer software based on reactions annotated in TRANSPATH®. The information about drugs corresponding to identified drug targets and clinical trials references were extracted from the HumanPSD^TM^ database (Wingender et al., 2007), release 2020.2^6^. The Ensembl database build 99.38^7^ (Aken et al., 2016) was used for gene ID representation and GO^8^ (Ashburner et al., 2000) was used for functional classification of the studied gene set.

### Functional Annotation

To explore the biological importance of gene signatures, a gene set enrichment analysis is performed. All the adjusted *p-*value significant genes were used. GSEA is an efficient method to determine whether the genes of interest show statistically significant enrichment between different biological states. GO enrichments for cellular component, biological process, and molecular functions were performed. To investigate the top enriched ontology terms, 1,000 random permutations were done and an adjusted *p*-value cutoff of 0.05 is used. The dysregulated gene network enrichment also gives a useful insight about known disease signatures (Subramanian et al., 2005). The hallmark gene set of MSigDB (Liberzon et al., 2011) defines specific biological states or processes. Enrichment analysis is performed in R using DOSE package (Yu et al., 2015). PANTHER pathway enrichment of the identified TFs was performed using the EnrichR tool (Chen et al., 2013). TRANSPATH^®^, (Krull et al., 2003) pathway enrichment was performed using the geneXplain platform.

### Genome Enhancer Pipeline

The approaches mentioned above help us in understanding the impact of the DEGs in GBM biology. To understand the reason behind this dysregulation, the genome enhancer pipeline of geneXplain is used. The genome enhancer is a multi-omics analysis service (see text footnote 1) that is built using an open-source programming environment BioUML (Kolpakov et al., 2019)^9^ and incorporates an automated pipeline for the previously published “upstream analysis” (Koschmann et al., 2015; Boyarskikh et al., 2018) and the advanced approach “walking pathways” (Kel et al., 2019). Significantly upregulated genes in STS were used in this workflow.

The workflow works in 2 steps.

A. Analysis of enriched transcription factor binding sites and composite modules

Binding of TFs to the specific sites in promoters and enhancers is the key to the transcriptional regulation of genes. Identifying clusters of binding sites for TFs (composite modules) in the upstream regulatory regions [−1,000 bp upstream of transcription start site (TSS)] of the genes of interest is a determining step to understand the gene regulatory mechanism (composite regulatory modules) (Kel-Margoulis et al., 2002).

We use the CMA (Waleev et al., 2006) to detect such potential enhancers, as targets of multiple TFs bound to the regulatory regions of the genes of interest. The TFs are ranked based on (a) the yes/no ratio: given a set of promoter sequences of dysregulated genes, denoted as a yes set, and promoter sequences of unchanged genes under the same experimental condition, denoted as a no set, motifs are considered important if they have a high yes/no ratio, the ratio of motif occurrences per promoter in yes and no sets, and a statistically significant enrichment of occurrences in yes sequences assessed by the binomial *p-*value. (b) A regulatory score, which is a measure of involvement of a TF in controlling the expression of genes that encode master regulators. CMA identifies the TFs that, through their cooperation, provide a synergistic effect and thus have a great influence on the gene regulation process.

B. Finding master regulators in networks

The second step involves the signal transduction database TRANSPATH^®^, and special graph search algorithms to identify common regulators of the revealed TFs. These master regulators appear to be the key candidates for therapeutic targets as they have a master effect on the regulation of intracellular pathways that activate the pathological process of our study. Master regulators regulating the TFs revealed in step A are ranked based on (a) logFC, (b) CMA score, which signifies how strong is the potential for this gene to be regulated by TFs of interest, and (c) master regulator score, which signifies how strong is the potential of this gene product to regulate the activity of those TFs. Selected master regulators can also be visualized and with the possibility to map the logFC and *p*-value on the created regulatory network.

### Validation of Observed Gene Signatures

The raw microarray data of 560 TCGA-GBM samples were downloaded from TCGA legacy. The GSE16011 raw.CEL data was downloaded from the GEO repository. Both raw datasets were processed and analyzed independently following same steps as mentioned earlier. These two datasets are used to observe and validate the expression pattern of master regulators across the two survival groups (see Table 6). GSE16011 comprises of data generated at a single center and is used in several studies (Prasad et al., 2020), unlike TCGA. TCGA-GBM microarray data PCA plots are given Supplementary Figure 3, and no significant batch effects in the context of survival groups were found.

**TABLE 6:** Statistics of the two validation datasets.

Datasets	Platform	Short-term survivors	Long-term survivors
GSE16011 (Gravendeel et al., 2009)	HU133 plus 2.0 arrays	93	16
TCGA-GBM microarray (Grossman et al., 2016)	HU133	271	49

### Validation of Master Regulators

The TCGA-GBM microarray data downloaded from TCGA legacy archive is processed in the same fashion as GSE. Similar cutoffs (log2FC and *p-*value) and parameters are used to identify enriched TFs and network analysis in order to understand drivers of gene regulatory networks in short survival.

### Impact on Survival

Master regulators and their target TFs affect the whole regulatory network and therefore can have an independent impact on survival in GBM patients. Level 3 RNA-seq data and clinical data for 152 TCGA-GBM cohort is downloaded using the TCGAbiolinks package in R. Survival and survminer libraries in R were used to perform a univariate survival analysis. A univariate survival analysis was used to understand the impact of individual master regulator on survival in GBM with non-overlapping 50% upper and lower quantiles. Additionally, a univariate Cox regression for survival analysis was performed using the coxph function of the survival package to calculate the HR with *p*-value cutoff of 0.05 for significance.

## Conclusion

In the work presented, we have identified candidate master regulators responsible for gene dysregulation in STS. These candidates have sufficient experimental evidence toward their role in GBM. Out of reported five master regulators, IGFBP2 is established as the most promising master regulator. Through the gene regulatory network analysis, we propose that IGFBP2 and FRA-1 are in a positive feedback loop that may lead to a pathological self-enhancing process responsible for poor survival in GBM.

## Data Availability Statement

The results of the analysis performed using the Genome Enhancer in geneXplain platform are available here: https://github.com/genexplain/Manasa_KP_et_al_IGFBP2_regulatory_networks_in_Gliobastoma.

## Author Contributions

AK involved in conceptualization, providing resources, supervision, and manuscript reviewing. MK had conceptualized the work, performed the data collection and data analysis, interpreted the results, and wrote the manuscript. DW supervised the data analysis pipeline and manuscript writing. EW and TB involved in supervision of work and in reviewing the draft. All authors contributed to the article and approved the submitted version.

## Conflict of Interest

MK, DW, and TB are from the Department of Medical Bioinformatics, University Medical Center Göttingen. MK, AK, and EW are employees of geneXplain GmbH. The remaining author declares that the research was conducted in the absence of any commercial or financial relationships that could be construed as a potential conflict of interest.

## Footnotes

1 https://genexplain.com/genome-enhancer/

2 https://www.ncbi.nlm.nih.gov/geo/query/acc.cgi?acc=GSE108474

3 https://www.ncbi.nlm.nih.gov/geo/query/acc.cgi?acc=GSE53733

4 https://genexplain.com/transfac

5 https://genexplain.com/transpath

6 https://genexplain.com/humanpsd

7 http://www.ensembl.org

8 http://geneontology.org

9 www.biouml.org
