# Supplementary material for: IGFBP2 Is a Potential Master Regulator Driving the Dysregulated Gene Network Responsible for Short Survival in Glioblastoma Multiforme
Source: Front Genet. 2021 Jun 15;12:670240. doi: 10.3389/fgene.2021.670240 (PMC8239365; doi:10.3389/fgene.2021.670240)
Supplement: Supplementary file 1 [file Data_Sheet_1.zip › Supplementary Tables & Figures.pdf]

Supplementary Materials

IGFBP2 is a potential master-regulator driving the dysregulated gene network responsible for short survival in Glioblastoma multiforme

Manasa KP, Darius Wlochowitz, Edgar Wingender, Tim Beißbarth, Alexander Kel

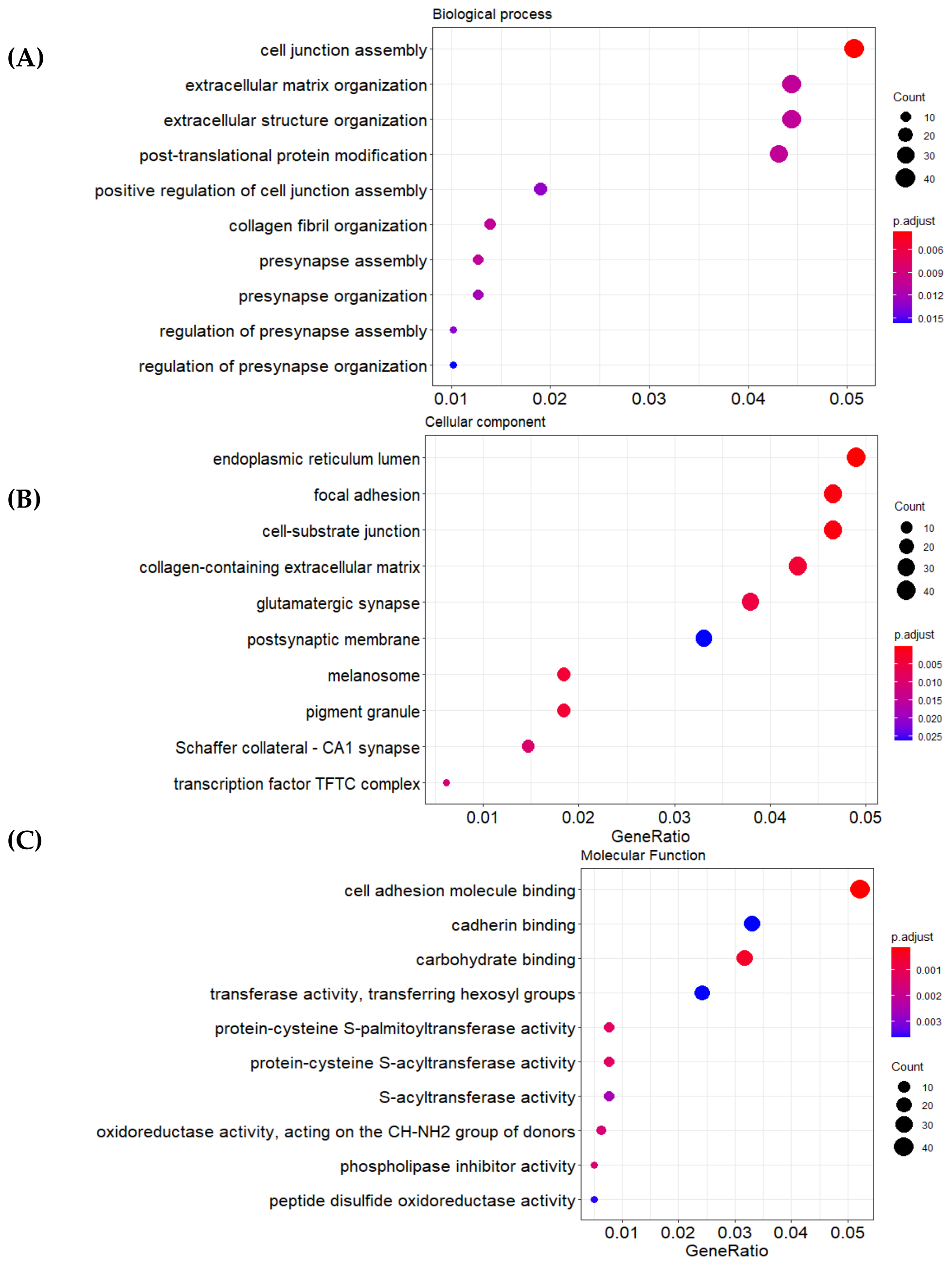

Figure 1. Functional enrichment analysis for significantly differentially expressed genes (adj.*p*-value <0.05) (A) Dot plot for enriched GO Biological Process. Y-axis represents enriched ontology categories and X-axis represents the GeneRatio. The Gene ratio is defined as count/set-size where 'count' is the number of genes that belong to a given gene set and 'set-size' is the total number of genes in the gene set. Y-axis is sorted based on the Leading-edge. Leading edge defines the subset of genes which contribute most to the Enrichment Score. The dots are sized based on gene ratio and are coloured according to their adj.*p*-value. (B) Enrichment for GO Cellular Components similar to A (C) Enrichment for GO Molecular Function similar to A and B.

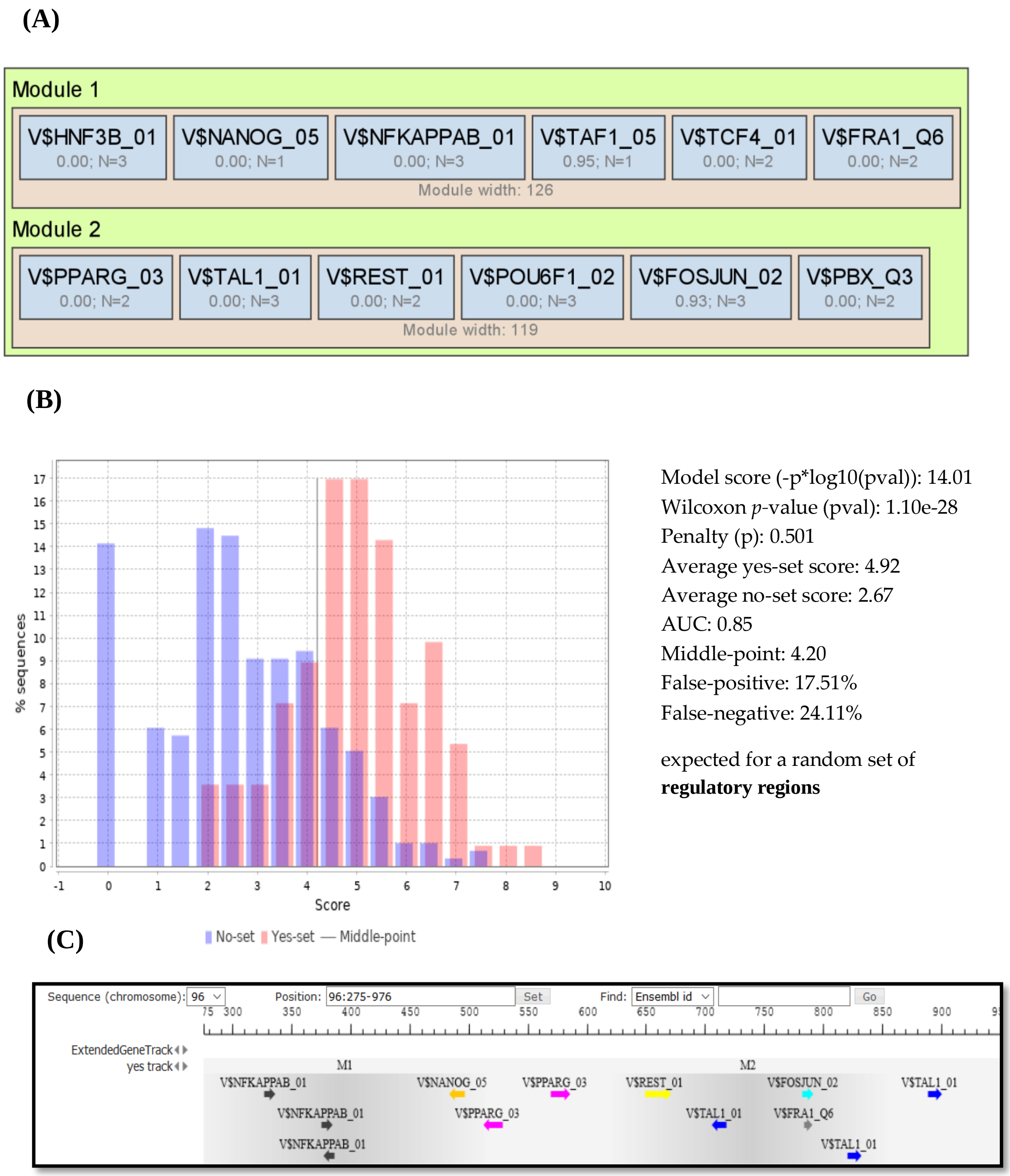

Figure 2. Results of CMA analysis of upregulated genes in short-term survivors. (A) Combination of 12 PWMs (position weight matrices) with their optimized cut-offs identified by genetic algorithm. A cut-off 0.00 reflects the algorithm chose the default cut-off; parameter N represent the maximal number of top scoring TF binding sites that are used in the module) (B) The discriminative parameters of the composition of the "Composite Score" (*p*-value of the Wilcoxon test, AUC, rates of false positives and false negatives) and two histograms of the distributions of the Composite Score values in "Yes" and "No" promoters. (C) An example of the site location in the promoter of CHI3L1 gene which is usually found upregulated in GBM. The promoter of the gene contains predicted sites for NFKB1, FRA1, NANOG and several other transcription factor

Table 2. Table of the transcription factors identified in the CMA analysis of the GSE dataset, log2FC (STS vs LTS), *p*-value and adj.*p*-value in STS across 3 datasets GSE, TCGA-GBM microarray and GSE16011.

Transcription factors	GSE			TCGA Dataset			GSE16011		
	log2FC (STS vs LTS)	*p*-value	adj.*p*-value	log2FC (STS vs LTS)	*p*-value	adj.*p*-value	log2FC (STS vs LTS)	*p*-value	adj.*p*-value
FOSL1	0.2353	0.00833	0.093832	0.022	0.866477	0.942691	0.461	0.032989	0.170584
PBX3	0.173	0.12895	0.372164	0.163	0.151272	0.411335	0.558	0.016908	0.113002
NFKB1	0.083	0.155435	0.408934	-0.004	0.956529	0.982133	0.209	0.115989	0.355865
PBX2	0.082	0.102006	0.33059	-0.22	0.001109	0.029677	-0.278	0.019596	0.12327
PPARG	0.0699	0.222946	0.491011	0.129	0.375068	0.643568	0.463	3.60E-04	0.008414
TAL1	0.027	0.384873	0.647536	-0.33	1.80E-05	0.003086	0.115	0.459738	0.692082
REST	0.021	0.578374	0.787431	0.176	0.028054	0.163735	0.288	0.034068	0.174094
RELA	0.007	0.90247	0.957246	0.072	0.281728	0.557129	-0.142	0.304378	0.576816
FOXA2	-0.05	0.336166	0.604469	-0.199	0.015938	0.120938	0.166	0.322979	0.592225
NANOG	-0.07	0.111174	0.344927	-0.189	0.038579	0.196264	-0.344	0.108064	0.344113
PBX1	-0.102	0.166715	0.42262	0.06	0.564011	0.77815	0.029	0.887276	0.94975
TCF7L2	-0.12	0.147798	0.399134	-0.084	0.406285	0.671782	-0.531	0.00498	0.051104

(A)

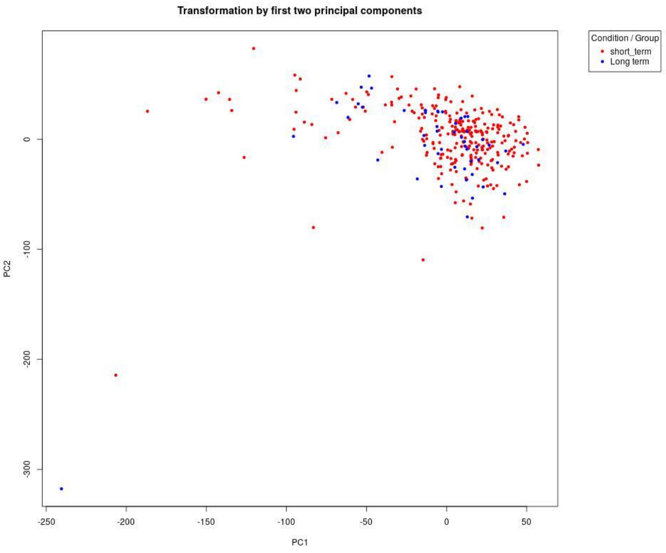

(B)

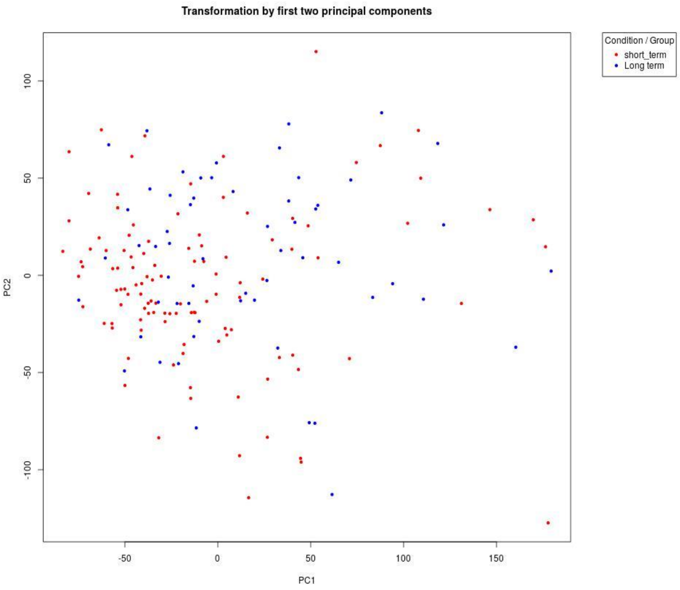

(C)

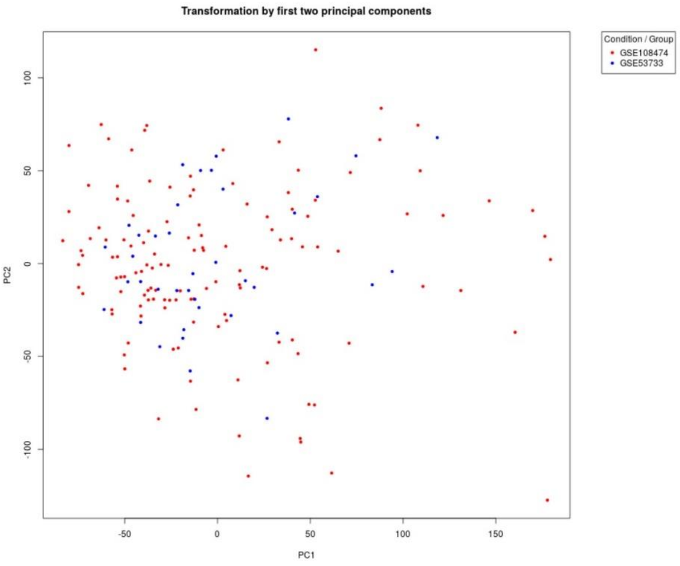

Figure 3. PCA plot for normalized (A) TCGA-GBM data (B) GSE (GSE108474, GSE53733) datasets show no significant batch effects across survival groups colored as red (STS) and blue (LTS) (C) PCA plot for normalized GSE data shows no batch effects between GSE108474 colored in red and GSE53733 colored in blue.

Table 3. The 16 Transcription Factors predicted from the CMA method that potentially regulate the genes upregulated in STS TCGA cohort (log2FC > 0.5 and adj.*p*-value<0.05)

Gene description	Gene symbol	Yes-No ratio	Regulatory score
E2F transcription factor 1	E2F1	1.249664	1.714286
LYL1 basic helix-loop-helix family member	LYL1	1.165219	0
nescient helix-loop-helix 2	NHLH2	1.165219	0
glial cells missing transcription factor 1	GCM1	1.294249	1.275613
high mobility group AT-hook 1	HMGA1	1.409148	1.295815
heart and neural crest derivatives expressed 1	HAND1	1.295599	0
nuclear receptor subfamily 3 group C member 1	NR3C1	1.388077	1.44531
TAL bHLH transcription factor 1, erythroid differentiation factor	TAL1	1.648248	1.88456
transcription factor 3	TCF3	1.500366	1.703319
HNF1 homeobox A	HNF1A	2.255652	1.043357
HNF1 homeobox B	HNF1B	1.178953	0
myeloid zinc finger 1	MZF1	1.300681	0
Kruppel like factor 4	KLF4	1.289327	1.492063
lymphoid enhancer binding factor 1	LEF1	2.299881	1.460317
glial cells missing transcription factor 2	GCM2	1.294249	0
high mobility group AT-hook 2	HMGA2	1.409148	1.212121

A)

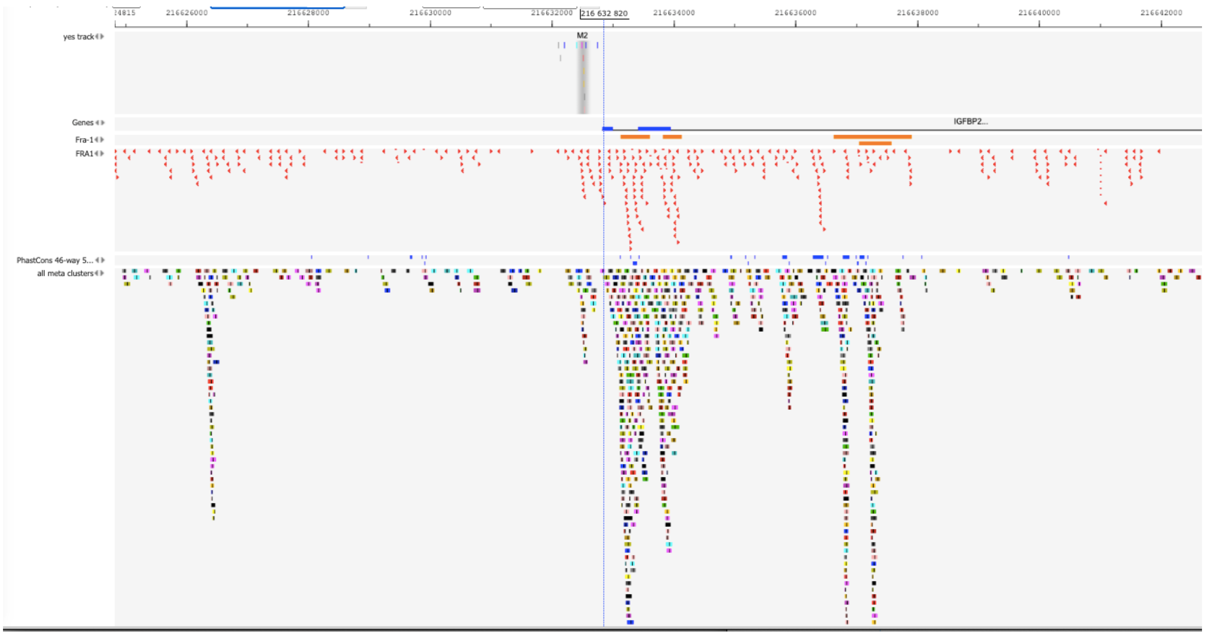

B)

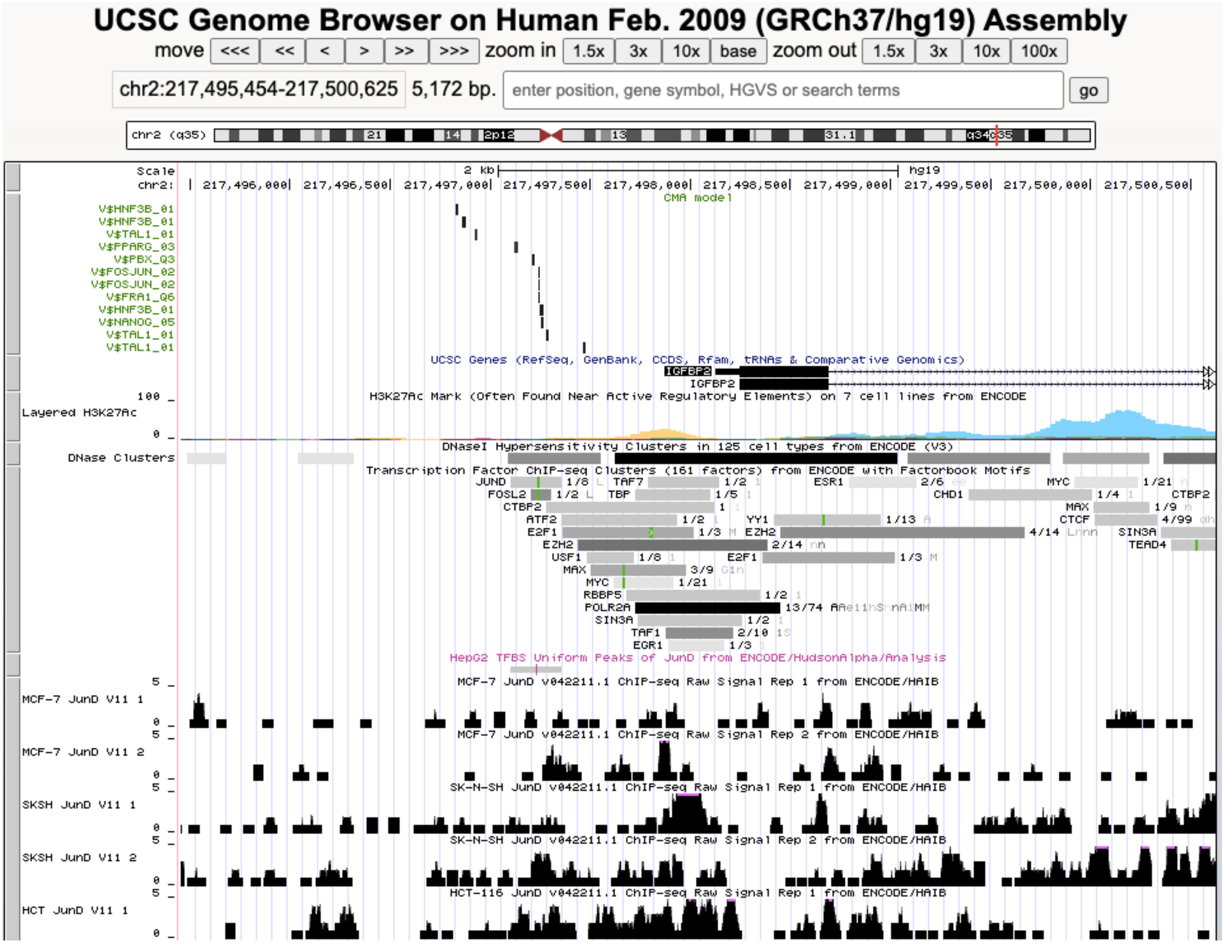
UCSC Genome Browser on Human Feb. 2009 (GRCh37/hg19) Assembly
chr2:217,495,454-217,500,625 5,172 bp.
CMA model
UCSC Genes (RefSeq, GenBank, CCDS, Rfam, tRNAs & Comparative Genomics)
IGFBP2
H3K27Ac Mark (Often Found Near Active Regulatory Elements) on 7 cell lines from ENCODE
Layered H3K27Ac
DNaseI Hypersensitivity Clusters in 125 cell types from ENCODE (V3)
DNase Clusters
Transcription Factor ChIP-seq Clusters (161 factors) from ENCODE with Factorbook Motifs
HepG2 TFBS Uniform Peaks of JunD from ENCODE/HudsonAlpha/Analysis
MCF-7 JunD v042211.1 ChIP-seq Raw Signal Rep 1 from ENCODE/HAIB
MCF-7 JunD V11 1
MCF-7 JunD v042211.1 ChIP-seq Raw Signal Rep 2 from ENCODE/HAIB
MCF-7 JunD V11 2
SK-N-SH JunD v042211.1 ChIP-seq Raw Signal Rep 1 from ENCODE/HAIB
SKSH JunD V11 1
SK-N-SH JunD v042211.1 ChIP-seq Raw Signal Rep 2 from ENCODE/HAIB
SKSH JunD V11 2
HCT-116 JunD v042211.1 ChIP-seq Raw Signal Rep 1 from ENCODE/HAIB
HCT JunD V11 1

Figure 4. Maps of regulatory element in upstream region and of IGFBP2 gene. A) Genome browser of geneXplain platform. Track "yes track" represents the cluster of identified TF binding sites of the composite model within the promoter. The track "FRA1" represents the mapped reads of the FRA1(HGNC symbol - FOSL1) ChIP-seq data from (https://www.ncbi.nlm.nih.gov/geo/query/acc.cgi?acc=GSM803382). The track "all meta clusters" shows all known meta-clusters in this region from GTRD database that represent the overlapping fragments of peaks for one particular TF from several ChIP-seq experiments. B) UCSC genome browser. Tracks with PWMs showing the positions of predicted TF binding sites of the composite model within the promoter of IGFBP2. There are separate tracks for limited set of ENCODE data about ChIP-seq peaks of TFs from Factorbook. Last five tracks represent the ChIP-seq signals of the JunD factors in various cell lines.

Table 4. Table of the master regulators identified, their description, log2FC in STS and number of TFs regulated.

Molecule Name	log2FC	CMA score	Reached from set
**FGFR3**	1.073	12.142	10
**AEBP1**	0.972	11.164	10
**IGFBP2**	1.098	10.191	10
**CNR1**	0.745	7.323	10
**TRIM22**	0.638	11.303	10
**SPRY2**	0.584	9.602	10
**CASP1**	0.524	8.102	10
**DUSP6**	0.705	8.742	10
**CXCL8,CXCR1,CXCR2,GNAI2**	1.050	10.031	10
**CD14,IRAK1,IRAK2,LBP,LY96,MYD88,TIRAP,TLR4**	0.741	8.277	10
**IL1B,IL1R1,IL1RAP,IRAK1,IRAK2,IRAK4,MYD88,TOLLIP**	0.950	9.157	10
**PDGFA**	0.825	7.948	10

Table 5. Table containing Yes/No ratio and corresponding *p*-value of enrichment for FRA1 transcription factor binding sites in GSE and TCGA dataset. There are 4 binding sites enriched for FRA-1 transcription factor and are all found to be significantly enriched in GSE and is validated in TCGA dataset.

ID	GSE dataset		TCGA dataset	
	Yes/No Ratio GSE	*p*-value_GSE	Yes/No Ratio TCGA	*p*-value_TCGA
V$FRA1_Q5	2.45536	0.00117	6.20968	5.8419E-5
V$FRA1_Q6	2.45536	0.00117	2.17735	3.9016E-6
V$FRA1_Q6_01	2.90434	4.1107E-4	2.47487	5.9147E-4
V$JUNBFRA1_01	2.60357	7.4892E-7	1.30521	2.1143E-6

Table 6. GBM subtypes across survival groups and across 4 datasets used in this study.

GBM Subtype	GSE108474		GSE53733		GSE16011		TCGA_GBM	
Verhaak_2010 subtyping	STS	LTS	STS	LTS	STS	LTS	STS	LTS
Classical	50	10	04	07	46	01	66	08
Mesenchymal	16	04	08	04	18	02	84	12
Proneural	13	14	04	14	13	13	67	07
Neural	18	07	00	00	16	00	41	21
Total	97	35	16	25	93	16	258	48

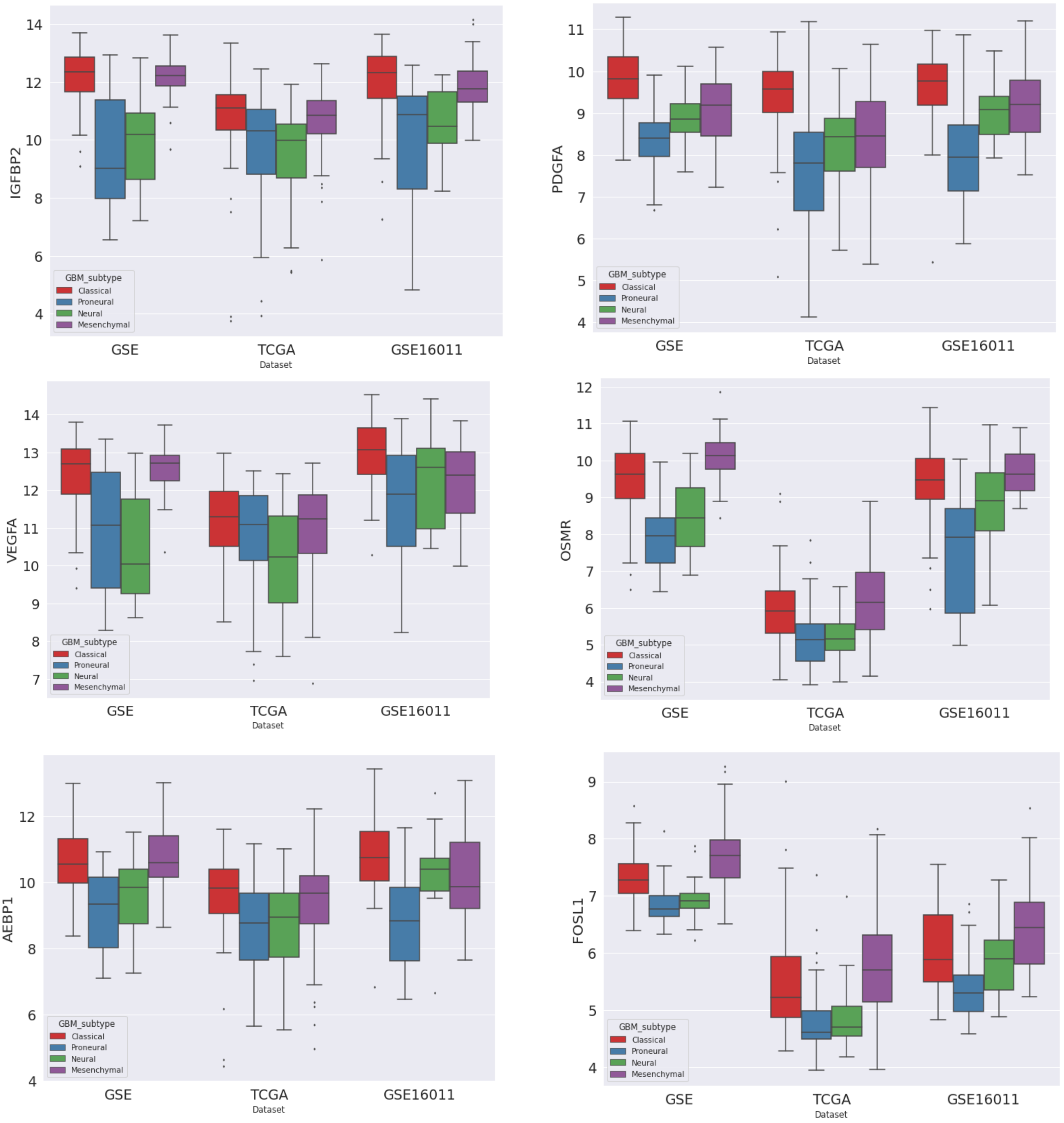

Figure 5. Expression levels of the five master regulators and FRA-1 transcription factor across subtypes of GBM in GSE, TCGA and GSE16011 cohorts.
